# Increased Incidence of Perforated Appendicitis in Children During COVID-19 Pandemic in a Bavarian Multi-Center Study

**DOI:** 10.3389/fped.2021.683607

**Published:** 2021-05-07

**Authors:** Frank-Mattias Schäfer, Johannes Meyer, Stephan Kellnar, Jakob Warmbrunn, Tobias Schuster, Stefanie Simon, Thomas Meyer, Julia Platzer, Jochen Hubertus, Sigurd T. Seitz, Christian Knorr, Maximilian Stehr

**Affiliations:** ^1^Department of Pediatric Surgery and Pediatric Urology, Cnopfsche Kinderklinik, Nuremberg, Germany; ^2^Department of Pediatric Surgery, Klinikum Dritter Orden, Munich, Germany; ^3^Department of Pediatric Surgery, Klinikum Schwabing, Technical University Munich, Munich, Germany; ^4^Department of Pediatric Surgery, University Hospital Augsburg, Augsburg, Germany; ^5^Department of Pediatric Surgery and Pediatric Urology, Klinikum Nürnberg, Nuremberg, Germany; ^6^Department of Pediatric Surgery, Pediatric Urology and Pediatric Trauma, Hospital for General, Visceral, Vascular and Pediatric Surgery, University Hospital Würzburg, Würzburg, Germany; ^7^Center for Pediatric and Adolescent Medicine, Children's Hospital St. Marien gGmbH, Landshut, Germany; ^8^Department of Pediatric Surgery, Dr. von Hauner Children's Hospital, University Hospital Munich, Munich, Germany; ^9^Department of Pediatric Surgery, University Hospital, Friedrich-Alexander-Universität (FAU) Erlangen, Erlangen, Germany; ^10^Department of Pediatric Surgery and Pediatric Orthopedics, Barmherzige Brüder Hospital – St. Hedwig Regensburg, Regensburg, Germany

**Keywords:** appendicitis, perforation, perforated appendicitis, COVID-19, pandemic

## Abstract

**Introduction:** Since early 2020 the COVID-19 pandemic and statutory preventive reorganization of treatment capacities with cancellation of elective surgery as well as curfew regulations led to vastly decreased utilization of primary health care.

**Materials and Methods:** To assess whether there are negative effects on pediatric acute care in Bavaria during the spring 2020 lockdown a state-wide retrospective multi-center study was performed to analyze the rate of perforated appendicitis during lockdown. Children who have been operated on during the corresponding period in 2018/19 served as control group.

**Results:** Overall, 514 patients (292 boys, 222 girls) were included (2020: 176 patients; 2019: 181 patients; 2018: 157 patients). Median age was 11.2 years. Four hundred thirty-nine patients (85.4%) underwent laparoscopic surgery, 69 (13.4%) open surgery and 1.2% underwent conversion from laparoscopic to open surgery. In 2020 a perforation rate of 27.8% (49/176 patients) was found, in 2018–2019 perforation rate was 20.7% (70/338 patients, *p* = 0.0359, Cochran-Mantel-Haenszel-Test). Subgroup analysis showed that in younger patients (≤ 11.2 years), in 2020 perforation rate was significantly higher with 37.6% (32/85 patients), while 22.2% (39/176) in 2018/2019 (*p* = 0.014, Fisher's exact test).In boys perforation rate was significantly higher in 2020 with 35.0% (35/100 patients) compared to 21.4% in 2018–2019 (*p* = 0.0165, Fisher's exact test).

**Conclusion:** During the period of curfew regulations in Bavaria the rate of perforated appendicitis in childhood increased significantly, especially in younger children and boys. Potentially this has to be attributed to delayed presentation to pediatric surgery care. Because of potential long-term sequelae of perforated appendicitis these adverse effects during curfew have to be taken into account for future political decision making to ensure reasonable patient care and avoid collateral damage in near-future or on-going pandemic situations.

## Introduction

In early spring 2020 the emergence of the COVID-19 pandemic (1) has led to dramatic and so far unprecedented challenges to the health systems worldwide. Since both the magnitude of the outbreak and statutory preventive measurements varied regionally to a large extent, the influence of these measures can be studied under different circumstances. In Bavaria, one of the largest states of Germany, a state-wide reorganization of available treatment capacities with the cancellation of elective surgery was ordered and curfew regulations (“lockdown”) were imposed on the general public beginning on March 20, 2020. This—and similar measures in other states of Germany—led to a vastly decreased utilization of primary health care in the emergency departments (2). This has been reported also in other regions with a much higher incidence of COVID-19 cases, such as New York, where in a single institution a reduction of pediatric emergency cases to 13% compared to the same period in the previous year was noted (3). Parental fear of seeking care during the pandemic is potentially contributing to this change, as well as the wish not to overburden the strained hospital resources with—seemingly—trivial health care problems. This behavior may lead to a severe impairment of overall public health in various fields, which has been termed “Corona Collateral Damage Syndrome” (4).

In this study we aim to investigate whether this is also the case in pediatric appendicitis, which is one of the most common emergency surgeries in (pediatric) surgery with about 100 per 100,000 patient-years in Europe and America with an age peak between 10 and 19 years (5).

Therefore, we conducted a Bavarian state-wide study including all major pediatric surgery institutions to evaluate the rate and clinical course of pediatric acute appendicitis during lockdown and assess potential collateral damage.

## Materials and Methods

After procurement of institutional ethics committee approval (ref. no.: 338_20 Bc) medical charts of all patients <18 years which were operated on because of suspected appendicitis during the lockdown phase in Bavaria were reviewed in all 10 participating departments. The time frame for the analysis was set to the period of March 20, 2020 (the beginning of the statutory curfew regulation in Bavaria) to May 31, 2020, when gradually lifting of the restrictive regulations had led to a near-normal state of affairs again. Patients which were operated on in the corresponding time periods of the previous years 2019 and 2018 were used as control group.

Data collection included sex, age, clinical, and histological description of the intraoperative findings, length of stay before and after the operation, the mode of surgery (open, laparoscopic, single-port, converted), length of antibiotic treatment, status of COVID-19 testing (if applicable) and need for re-operation.

The patients were grouped into four different groups depending on the intraoperative and histologic findings: (a) subacute/negative, if clinical and histopathologic both reports revealed no relevant appendicitis; (b) acute to gangrenous, if a varying degree of appendicitis was noted, but no perforation either in clinical or pathologic report, (c) Perforated appendicitis if either documentation by the attending surgeon or the description of perforation in the histopathologic report stated perforation. This was defined as primary outcome measure. In group (d) intraoperative findings not fitting in any of the other groups were collected (e.g., oxyuriasis, neuroendocrine tumor of the appendix). Since it's a well-known fact that in acute appendicitis there is only weak concordance between perioperative diagnosis and histopathology report, we decided not to distinguish non-perforated appendicitis into additional subgroups such as phlegmonous or gangrenous appendicitis (6, 7). If there was discordance between clinical description and histopathology report, the worse of the two was used for our study classification (e.g., if intraoperative finding was termed “subacute appendicitis” and histopathologic description was “phlegmonous appendicitis” the latter was used to group the case.

Microsoft Excel 365® (RRID:SCR_016137) was used for data collection, statistical analysis was performed using GraphPad Prism®, RRID:SCR_002798) version 7.0 (San Diego, USA) using Fisher's exact test/chi-square test, one-sided ANOVA and Cochran-Mantel-Haenszel-test. ReviewManager® (RevMan, RRID:SCR_003581) version 5.4.1 was used to create the Forest plot. Statistical significance was assumed at *p* < 0.05. Odds ratios (ORs) and 95% confidence intervals (CIs) were calculated for comparisons.

## Results

During the curfew period in 2020 we identified 176 patients which were operated on because of acute appendicitis across the 10 participating institutions. In the previous years during the same period there were 181 patients in 2019 and 157 in 2018. Basic demographic data is presented in Table 1.

**Table 1:** Basic demographic data of all patients with acute appendicitis during the CoVID-19 curfew and control groups 2018–2019.

	**2018** ***n* = 157**	**2019** ***n* = 181**	**2018–2019** ***n* = 338**	**2020** ***n* = 176**	***p***
**Sex**					
Female	62 (39.5%)	84 (46.4%)	146 (43.2%)	76 (43.2%)	
Male	95 (60.5%)	97 (53.6%)	192 (56.8%)	100 (56.8%)	0.43^a^
Mean age (years)	11.3	11.1	11.2	11.2	0.91^a^
Range (years)	2.8–17.9	1.5–17.9	1.5–17.9	3.0–17.9	

a *One-sided ANOVA*.

No significant difference in the demographic data of the different groups could be identified. Therefore, the control groups of 2018 and 2019 were combined for further analysis.

During the study period, no pediatric patient with COVID-19 infection could be identified. However, because of limited testing resources and state policy especially during the first weeks of the study period, only 39.8% (70 of 176) of the patients had been tested preoperatively.

The number of patients included per institution varied between 7 and 33 during the COVID-19 period and between 10–36 and 8–28 in 2019 and 2018, respectively. Total number of patients per institution ranged from 33 to 97. The summary of clinical outcome is shown in Table 2.

**Table 2:** Primary and secondary outcome of patients with acute appendicitis during the study period 2020 and the combined control period 2018–2019.

	**2020**	**2019–2018**	***p***
	***n* = 176**	***n* = 338**	
**Operation on …**			
… admission day	118 (67.0%)	185 (54.7%)	**0.0081**^a^
… day after admission	43 (24.4%)	133 (39.3%)	
… 2 days or more after admission	15 (8.5%)	20 (5.9%)	
**Severity of appendicitis**			
*Perforated*	*49 (27.8%)*	70 (20.7%)	**0.035**^**b**^
*Acute/phlegmonous to gangrenous (non-perf.)*	*119 (67.6%)*	252 (74.6%)	
*Negative/subacute*	*6 (3.4%)*	13 (3.8%)	
*Other finding*	*2 (1.1%)*	3 (0.9%)	
Laparoscopic to open surgery conversion rate	3 (1.7%)	3 (0.9%)	0.42^a^
Mean length of stay (d) *Range (d)*	5.3 *2–17*	4.9 *2–20*	0.07^a^
Antibiotics given (%)	145 (82.4%)	279 (82.5%)	>0.99^a^
*If so, length of AB therapy (d)* *Range (d)*	4.4 *1–15*	3.8 *1–45*	
Reoperations (%)	4 (2.2%)	6 (1.8%)	0.74^a^

a *Fisher's exact test.*

b *Cochran-Mantel-Haenszel-test.*

*Bold indicate to highlight statistically significant differences. Italics indicate to show these lines are subitems to the lines before*.

**Figure 1:**
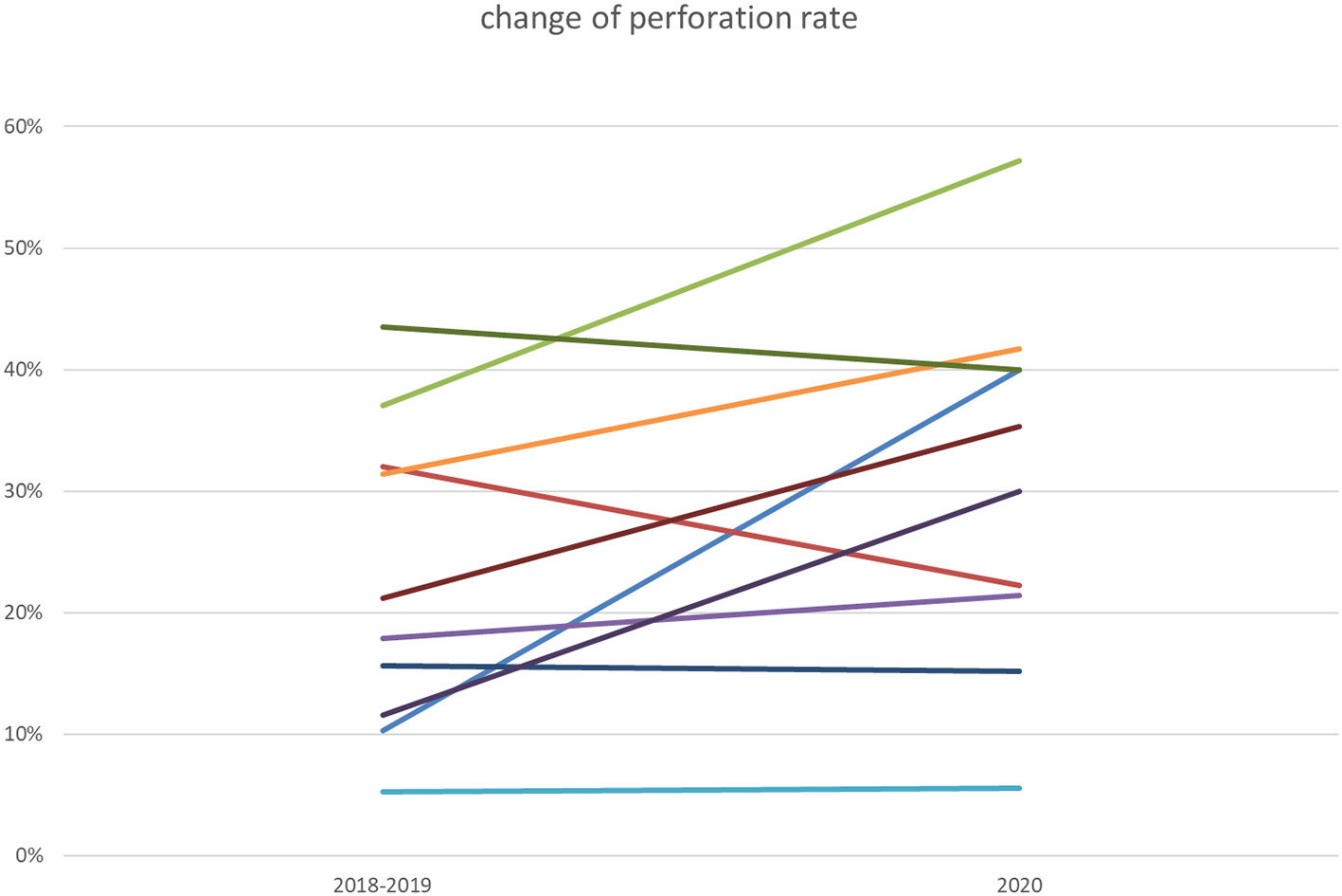
Change of perforation rate 2020 compared to previous years: Seven of ten participating centers noted an increase of perforation rates.

**Figure 2:**
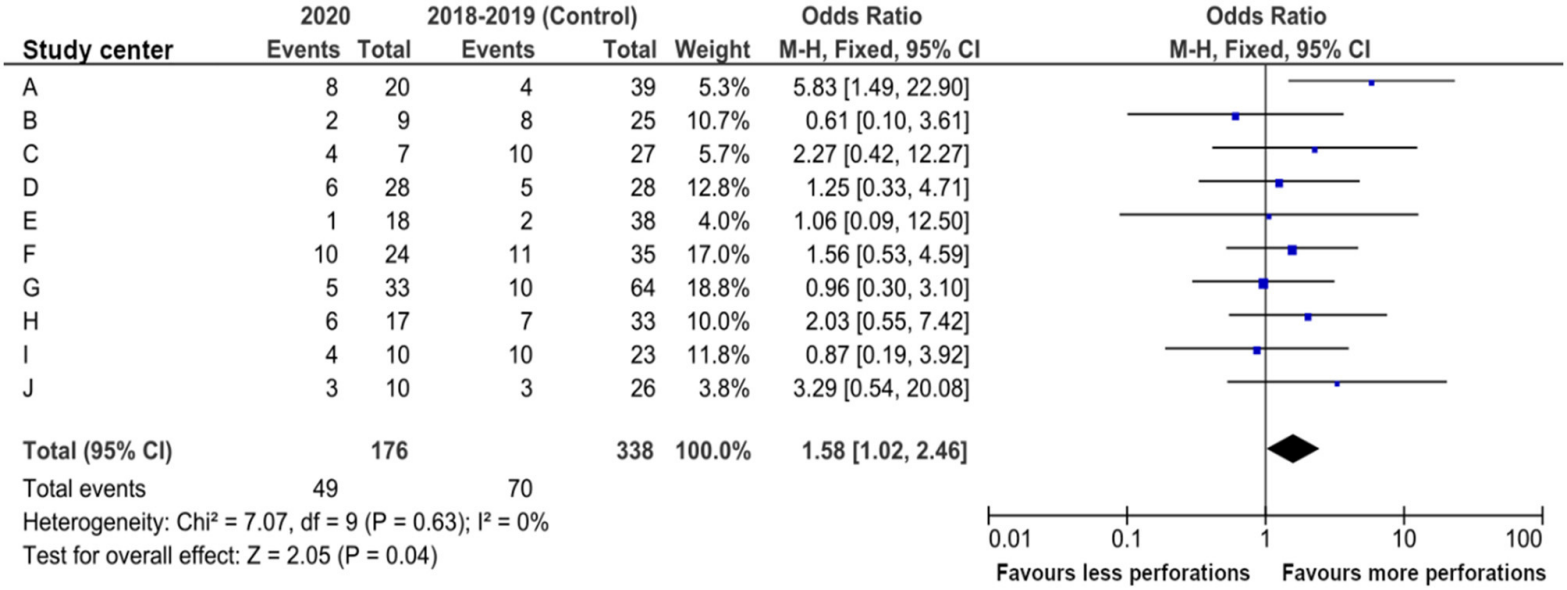
Forest plot of Cochran-Mantel-Haenszel test with details of study centers for perforated appendicitis per institution 2020 compared to the combined control period 2018–2019 (OR, odds ratio; CI, confidence interval).

**Figure 3:**
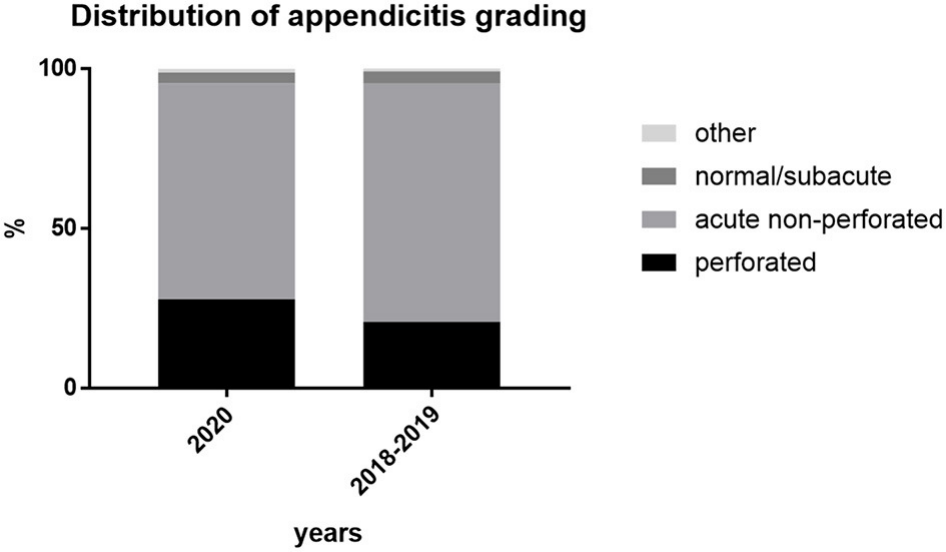
Grading of appendicitis in % of cases per group.

Overall perforation rate was 27.8% (49 of 176 patients) in 2020 (5.6–57.1% within the different institutions) and 70 of 338 patients (20.7%) in 2018–2019 (5.3–43.0%) (Figure 1), which was significant when compared to 2020 (*p* = 0.0359, OR 1.58, 95% CI 1.02–2.46; Cochran-Mantel-Haenszel test, Figure 2). Comparison of appendicitis grading based on the above-mentioned distinction (Figure 3 and Table 2) showed that the rate of negative appendectomy as well as findings other than appendicitis (oxyuriasis, neuroendocrine tumors) did not differ within the years.

**Figure 4:**
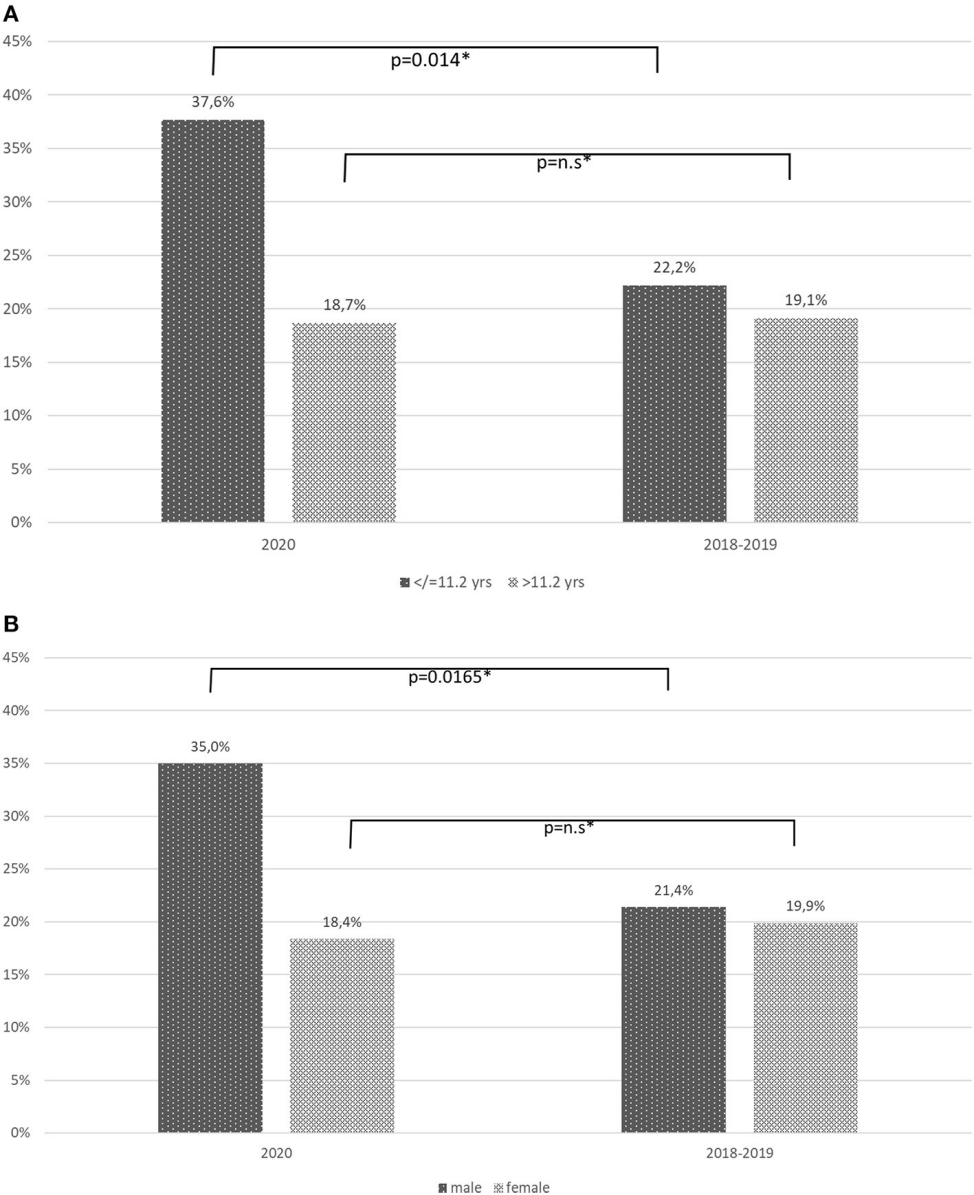
**(A)** Perforation rate depending on age groups shows increase of overall perforation rate is solely due to increased perforation rate in younger patients (*Fisher's exact test). **(B)** Perforation rate depending on sex shows increase of overall perforation rate in boys is significantly higher in 2020 (*Fisher's exact test).

For subgroup analysis the patients were divided in two age groups (< /=11.2 years and >11.2 years), according to the mean age of the study population. In the group with the younger patients, in 2020 perforation rate was 37.6% (32/85 patients), while 22.2% (39/176) in 2018/2019 (*p* = 0.014, Fisher's exact test). In older patients >11.2 years perforation rate was 18.7% (17/91 patients) in 2020 and 19.1% (31/162 patients) in 2018/2019 (*p* = n.s., Figure 4A). Subgroup analysis for sex showed that in boys perforation rate was significantly higher in 2020 with 35.0% (35/100 patients) compared to 21.4% in 2018–2019 (*p* = 0.0165, Fisher's exact test). In girls, no significant difference could be noted (Figure 4B).

Length of stay was 4.9 days (±0.123) during the control periods and 5.3 days (±0.198) in 2020 (*p* = 0.06; Table 2).

To rule out that the higher perforation rate in 2020 was due to delayed surgery after admission we determined the rate of patients who were operated on at day 0 (admission day), day 1 or later (Table 2). The data showed that the percentage of patients operated on admission day was higher than in previous years (67.0 vs. 54.7%, *p* = 0.0081, Fisher's exact test). For patients with perforated appendicitis alone, this rate was 77.6% in 2020 (38 of 49 patients) and 72.9% in 2018–2019 (51 of 70 patients, *p* = 0.67, Fisher's exact test).

Mode of surgery: The rate of laparoscopic surgery increased during the years: In 2018, 123 of 157 patients (78.3%) were operated on laparoscopically, which increased to 157 of 181 patients (86.7%) in 2019 and 159 of 176 patients (90.3%) in 2020. No patient was treated by primary abscess drainage. Conversion rate from laparoscopic to open surgery was overall low in all groups (1.8% in 2020, 1.1% in 2018–2019, *p* = 0.67, Fisher's exact test) and showed no significant difference. Likewise, the need for re-operation was low and not statistically different (2.2% in 2020 vs. 1.8% in 2018–2019, *p* = 0.74, Fisher's exact test, cf. Table 2). Reasons for re-operation in 2020 were intraabdominal abscesses in two cases, ileus and concomitant ovarian cyst in one case each. In 2018 and 2019 reasons were intraabdominal abscess formation in four cases, wound infection and post-operative bleeding in one case each.

## Discussion

The available data regarding the influence of COVID-19 associated curfew measures on pediatric appendicitis is far from conclusive with some centers reporting an increase of pediatric and adult appendicitis during the corona pandemic (8) while others showed a marked decrease (9, 10) or no significant changes (11). Already early on during the pandemic the first report of a delayed diagnosis in a small group of pediatric patients with complicated appendicitis was published (12).

In our study we could show that during the spring wave of COVID-19 there was a significantly higher proportion of perforated appendicitis in Bavarian children (27.8%) compared to the previous years while the rate of acute/uncomplicated appendicitis decreased at the same time and the overall number of patients remained stable.

Similar results have been reported from other geographic areas: In a single-center study on adult appendicitis in Argentina, a statistically significant increase in complicated appendicitis compared to the previous years (47 vs. 17%) has been found (13). In a study from Australia, the rate of complicated appendicitis increased from 24.6% in 2019 to 47.9% in 2020 (14). Also, in a recent global survey regarding the treatment of acute appendicitis during the pandemic, 34.2% of the participating surgeons asserted that patients presented with more advanced stages of the disease (15). The only other multi-center study available regarding the rate of pediatric perforated appendicitis originates from New York City Metropolitan region, an area with an extremely high incidence of COVID-19 and hence a crushing hospital burden (8): Similar to our study they noted a 45.5% decrease in uncomplicated appendicitis together with a 21.1 and 29% increase in perforated and gangrenous appendicitis, respectively. They could show that there was no difference in either the severity of appendicitis or the treatment course between COVID-19 positive vs. negative children. In our study, the finding that no children were identified which were positive to COVID-19 is concordant with data that hospitalizations of COVID-19-positive children in Germany were very rare during the spring pandemic, albeit only less than half of the patients were tested preoperatively, since routine testing of all patients was introduced only later in the year (16).

An early single-center report from the USA found a diminished number of overall appendicitis patients while noting an increased rate of complicated appendicitis (17). In contrast, Kvasnovsky et al. described an unchanged number of pediatric appendicitis cases in a single tertiary referral center during COVID-19 pandemic (18). In our data we could not detect a significant state-wide decrease in overall acute appendicitis patients during the study period. However, in a recent study using data from the largest German insurance provider (AOK), a significant reduction in overall numbers of both adult and pediatric appendectomies during spring lockdown in 2020 was noted. This decrease affected only appendectomies due to acute simple and non-acute appendectomies. Numbers for appendectomies in acute complex appendectomies remained unchanged. Female patients in the age group 1–18 years showed the strongest decrease (19). While this decrease could not be shown in our study, subgroup analysis for age and sex showed that the increase in perforation rate was solely due to an increase in younger patients (<11 years) and boys. This is not unexpected because of the possibility of unusual presentation common in younger children, which can lead to delay and therefore increased morbidity in appendicitis (20). A further potential explanation for the differences between the study by Maneck et al. and our data could be that in Bavaria lockdown measures beginning on March 20, 2020 were stronger than in other states, e.g., by imposing penalties and high monetary fines for violations of lockdown policy. This may have contributed to a different behavior of the population and therefore to regional differences in both spread of the disease as well as negative side effects of the lockdown measures, which are not apparent if looking on federal data (21).

The finding of an elevated perforation rate in 2020 is corroborated by a tendency to a longer length of stay in this year, although not statistically significant. A possible confounding factor which counterbalances the length of stay may well be a tendency to earlier dismissal in 2020 compared to the control group years due to either parents' or surgeons' wishes to ease hospital workload in the face of expected COVID-19 cases. However, no objective data is available to confirm this.

We believe that the decrease in utilization of primary healthcare as noted during the pandemic has led to a delay in presentation of children with appendicitis and therefore to an increased rate of perforation especially in younger children. It is known that infrequent health care utilization in general is associated with higher odds of perforated appendicitis in children. This association correlates with visit frequency in the year before presentation, even when adjusted for socio-economic background (22). It seems probable that similar effects are to be expected, if—through statutory measures and explicit recommendations toward the public—the overall frequency of primary health care visits of parents with children is reduced as was the case in the first wave of COVID-19 pandemic (2).

In the study by Fisher et al. a longer symptom duration of patients with perforation was noted (71 vs. 47 h in the control period), hinting to a delayed presentation (8). One other study found not difference in overall duration of symptoms in days during shelter-in-place orders 2020 compared to 2019 (23); however, since appendicitis is a rapidly progressing disease a measurement in days does not reflect the rapid course of the disease. Because of the retrospective nature of our study, duration of symptoms in hours was not available throughout the medical records and therefore could not be included in the analysis.

There are several further strengths and weaknesses to our study. One strength is the determination of start and end date of the study and control groups: First, the start date was chosen as the beginning of the state-wide lockdown measures which were imposed on a short notice and therefore communicated widely through multiple media types (including traditional media such as TV and social media). Thus, this probably influenced population behavior in a stronger degree than e.g., the day of the occurrence of the first case of COVID-19 in a given country (to which a large proportion of the population may not even have been aware of at that time) and which has been chosen in other studies (9). Second, the selection of control groups in the same time frame during the previous years helps to eliminate seasonal variation in acute appendicitis (24).

On the other hand, the relatively small duration of the study and control period (roughly 6 weeks each) leads to a relatively small number of patients per institution and therefore presents challenges to statistical significance. However, we believe that because of this limitation results are rather under- than overestimated. One possibility to further increase patient numbers at least in the control groups would have been to include more historical data from pre-2018. However, we opted against this, because longstanding trends in abdominal (pediatric) surgery, such as tendencies to reduce antibiotic treatments and an increase in laparoscopic surgery might disguise or distort our study results (25, 26). Also, the study is necessarily retrospective in nature, since no contemporary study group is available.

One other weakness of the study is the inherent difficulty of standardized grading of appendicitis and the low concordance between perioperative description by the surgeon and histopathology findings. Additionally, histopathologic examination is not standardized and may vary from hospital to hospital (27). There is not even consensus whether histopathologal or clinical description (which will influence post-operative treatment more than the pathology report) should be considered gold standard (6, 28). Thus, an amalgamated grading system as mentioned above seemed most reasonable for the study purpose. Another weakness is that data was only procured from pediatric surgery departments while an unknown number of children were probably also operated on in adult surgery departments.

Several changes to standard treatment of appendicitis have been proposed during the COVID-19 pandemic: First, the reversion from laparoscopic surgery to open surgery for appendicitis has been argued as potentially beneficial because of the possible reduction in aerosole production and therefore reduced risk of virus transmission (15). Therefore, despite low evidence, in some regions especially in the first phases of the pandemic, rates of open appendectomies (in adults) have increased during the pandemic (29). In our study, however, this phenomenon could not be noted. Rate of laparoscopic surgery continued to increase in accordance with recent trends from the literature (26).

Second, in areas of diminished operative capacities due to high numbers of COVID-19 patients a conservative approach to appendicitis in situations of overwhelming strain on the health system has been advocated (30–32). Nevertheless, data from NYC metropolitan region showed that even in regions with crushing hospital burden it was still possible to commit to surgical care of pediatric patients during the COVID-19 surge (8). Fortunately, this situation did not emerge in Bavaria during the Spring 2020 pandemic wave, where operative capacities for emergency cases were sufficient throughout the study period. This is supported by the time of appendectomy after admission: Patients with appendicitis did not show a longer preoperative delay in hospital compared to the previous years. Overall rate of operation on admission day increased significantly, which can be attributed to the increased severity but also potentially to under-utilized hospital capacities with a higher availability of operation theater time.

The German Society of Pediatric Surgery accordingly stated that emergency and exigent surgery should not be postponed in any case. Therefore, no different approach in the management of appendicitis was adopted in the participating centers. Surgical treatment of acute appendicitis in childhood should remain gold standard except in the extreme situation of non-accessibility of the operating room or the necessary staff (5, 33). Future attention should be directed to possible differences in the later course of the pandemic to assess the effect of specific influencing factors, such as changing curfew regulations or different behavior during later pandemic waves. In the last months during the further course of the pandemic, both federal and state-driven legislation has led to a rapidly changing and overlapping mixture of restrictions in Bavaria, which leads to great difficulties in assessing these factors, and this evolving situation is still on-going.

## Conclusion

During the period of curfew regulations in Bavaria the rate of perforated appendicitis in childhood and adolescence increased significantly, especially in younger and male patients. Potentially this has to be attributed to a delayed presentation to pediatric or pediatric surgery care due to the “stay-at home” policy, parent's fear of the hospital environment or the wish not to strain hospital workload further with seemingly manageable conditions. Because of potential long-term sequelae of perforated appendicitis such as intestinal adhesions with obstruction these adverse effects of a reduced utilization of health care facilities during curfew should be considered for future political decision making: To avoid collateral damage in near-future or on-going pandemic situations, it is important to advise legal authorities not to discourage the population to seek timely medical attention in case of emergency conditions.

## Data Availability Statement

The raw data supporting the conclusions of this article will be made available by the authors, without undue reservation.

## Ethics Statement

The studies involving human participants were reviewed and approved by Ethikkommission der Friedrich-Alexander-Universität (FAU) Erlangen, Krankenhausstr. 12, 91054 Erlangen, Germany. Written informed consent from the participants' legal guardian/next of kin was not required to participate in this study in accordance with the national legislation and the institutional requirements.

## Author Contributions

F-MS designed the study, collected data, analyzed the data, and drafted and revised the paper. JM, SK, JW, TS, SS, TM, JP, JH, STS, and CK collected data and drafted and revised the paper. MS designed the study, and drafted and revised the paper. All authors contributed to the article and approved the submitted version.

## Conflict of Interest

The authors declare that the research was conducted in the absence of any commercial or financial relationships that could be construed as a potential conflict of interest.
